# Factores pronósticos en pacientes hospitalizados con diagnóstico de infección por SARS-CoV-2 en Bogotá, Colombia

**DOI:** 10.7705/biomedica.5764

**Published:** 2020-11-13

**Authors:** Juan Camilo Motta, Danny Novoa, Carmen Cecilia Gómez, Julián Moreno, Lina Vargas, Jairo Pérez, Henry Millán, Álvaro Ignacio Arango

**Affiliations:** 1 Escuela de Medicina y Ciencias de la Salud, Universidad del Rosario, Bogotá, D.C., Colombia Universidad del Rosario Universidad del Rosario BogotáD.C Colombia; 2 Servicio de Medicina Interna, Fundación Cardioinfantil, Bogotá, D.C., Colombia Fundación Cardioinfantil BogotáD.C Colombia; 3 Servicio de Infectología, Fundación Cardioinfantil, Bogotá, D.C., Colombia Fundación Cardioinfantil BogotáD.C Colombia

**Keywords:** infecciones por coronavirus, síndrome respiratorio agudo grave, mortalidad, pronóstico, pacientes internos, Coronavirus infections, severe acute respiratory syndrome, mortality, prognosis, inpatients

## Abstract

**Introducción.:**

La infección por el nuevo coronavirus SARS-Cov-2 es una emergencia de salud pública en todo el mundo; su diagnóstico se basa en pruebas moleculares, en tanto que su pronóstico depende de los antecedentes del paciente y de algunos exámenes paraclínicos. En Colombia aún no se cuenta con datos de pronóstico en una población local.

**Objetivo.:**

Evaluar los factores asociados con el desarrollo de la enfermedad grave en pacientes hospitalizados con diagnóstico de infección por SARS-CoV-2, así como los factores pronósticos de la mortalidad.

**Materiales y métodos.:**

Se hizo un estudio de cohorte ambispectivo en pacientes hospitalizados en la Fundación Cardioinfantil entre marzo y junio de 2020.

**Resultados.:**

De los 104 pacientes analizados, en el 31,7 % (n=33) la infección fue grave y en el 9,6 % (n=10) se produjo la muerte. El factor pronóstico más importante de la mortalidad fue el desarrollo de la enfermedad grave, seguido de una edad de más de 60 años y la desnutrición. Para el desarrollo de la enfermedad grave los factores pronósticos fueron los antecedentes de hemodiálisis *(hazard ratio,* HR=135), diabetes (HR=4,4) y el aumento en el nivel de la lactato deshidrogenasa (LDH) (HR=1,004), en tanto que un conteo de linfocitos superior a 1.064 fue un factor protector (HR=0,9). El puntaje del *National Early Warning Score* (NEWS2) correspondiente a las categorías de alto y bajo riesgo fue el que mejor rendimiento tuvo. No hubo diferencia entre los tratamientos administrados.

**Conclusiones.:**

Los factores pronósticos más importantes para la mortalidad fueron tener más de 60 años, hipertensión, diabetes y cirrosis, en tanto que para el desarrollo de la enfermedad grave fueron la enfermedad renal crónica con hemodiálisis, un puntaje de NEWS2 de alto riesgo al ingreso, y aumento en los niveles de LDH y proteína C reactiva, y leucocitosis.

## Abstract (en)

**Introduction:**

Infection with the new SARS-Cov-2 coronavirus is a worldwide public health emergency; its diagnosis is based on molecular tests, while its prognosis depends on the patient's history and on some paraclinical tests. In Colombia, forecasts are not yet counted.

**Objective:**

To assess the factors associated with the development of severe disease in hospitalized patients diagnosed with SARS-CoV-2 infection, as well as the prognostic factors for the outcome of mortality.

**Materials and methods:**

We conducted an ambispective cohort study in hospitalized patients at the *Fundación Cadioinfantil* from March to June, 2020.

**Results:**

Of the 104 patients analyzed, 31.7% (n=33) had a severe presentation and 9.6% (n=10) had a mortality outcome. For mortality, the most important prognostic factor was the development of severe disease followed by age over 60 years and malnutrition. For the development of the severe disease, prognostic factors were a history of hemodialysis (HR=135), diabetes (HR=4.4), and an increased level of lactate dehydrogenase (LDH) (HR=1,004), while the lymphocyte count over 1,064 was a protective factor (HR=0.9). In the classification of patients, the National Early Warning Score (NEWS2) score in the high and low-risk categories corresponded to the best performance. There was no difference between the treatments administered.

**Conclusions:**

The most important prognostic factors for mortality were being over 60 years of age, hypertension, diabetes, and cirrhosis, while for the development of severe disease they were chronic kidney disease with hemodialysis, NEWS2 with high risk at admission, increased levels of LDH and C reactive protein (CRP), and leukocytosis.

La enfermedad infecciosa causada por el nuevo coronavirus SARS-Cov-2 (COVID-19), descrita inicialmente en diciembre de 2019 en Wuhan, capital de la provincia de Hubei (China) ^1^^,^^2^, ha tenido una rápida expansión mundial: hasta el 14 de septiembre de 2020 en 188 países ubicados en los cinco continentes se habían contabilizado 28'637.952 contagiados y 917.417 muertos ^3^. Hasta el 15 de septiembre del 2020 en Colombia se habían reportado 728.590 contagios y 23.288 muertes ^4^.

El nuevo coronavirus, denominado SARS-Cov-2 por el Comité Internacional de Taxonomía de Virus ^5^, es un virus envuelto de cadena sencilla y ARN en sentido positivo perteneciente al género *Betacoronavirus* de la familia Coronaviridae del orden Nidoviral ^6^^-^^8^. Está constituido por una envoltura de membrana con una bicapa lipídica y proteínas estructurales como la proteína de membrana (M), la proteína de envoltura (E) y la proteína *spike* (S), y un ARN de cadena sencilla unido a una nucleocápside ^8^^,^^9^.

La infección por SARS-Cov-2 produce la COVID-19, enfermedad que tiene un periodo de incubación medio de 5,2 días con un percentil 95 de 12,5 días ^2^. Entre los síntomas que presentan los pacientes, los más importantes son la fiebre (90 %), la tos (70 %), la disnea (40 %), las mialgias (25 %) y la producción de esputo (27 %), además de síntomas gastrointestinales como diarrea y emesis (17-27 %) ^1^^,^^2^^,^^10^^,^^11^.

La mayoría de los pacientes cursa con una enfermedad leve (81 %), pero el 14 % desarrolla la enfermedad moderada, el 5 %, la enfermedad grave y el 2,3 % muere ^2^. Entre los factores pronósticos descritos para el desarrollo de la enfermedad crítica y mortalidad, los más importantes son la edad, ya que se ha reportado una mortalidad del 8 % en pacientes de 70 años y del 14,8 % en pacientes de más de 80 años ^12^, y la presencia de ciertas comorbilidades como hipertensión arterial, diabetes, enfermedad pulmonar obstructiva crónica, enfermedad coronaria, asma, obesidad y antecedentes de tabaquismo activo ^10^^,^^13^^,^^14^. Asimismo, los resultados de los exámenes de laboratorio relacionados con las peores consecuencias son la elevación del dímero D (con punto de corte de 1 ng/ml), linfopenia (<800), niveles elevados de lactato deshidrogenasa (LDH) (>350 UI/L) y ferritina (>1.000), y prueba de troponina positiva ^10^^,^^14^^,^^15^.

Considerando los datos de los estudios internacionales realizados hasta la fecha, es importante contar con datos epidemiológicos sobre el comportamiento de la infección a nivel local y nacional. En ese sentido, en el presente estudio se planteó el objetivo de describir las características de nuestra población cautiva como las comorbilidades ^10^^,^^15^ y los síntomas en el momento del ingreso para evaluar el comportamiento clínico previo a la hospitalización, así como variables paraclínicas ya establecidas como factores pronósticos y hallazgos en las imágenes diagnósticas y su posible relación con los resultados clínicos de mortalidad en nuestro medio y bajo las condiciones sociodemográficas locales.

## Materiales y métodos

Se llevó a cabo un estudio observacional analítico de cohorte ambispectivo en adultos hospitalizados en la Fundación Cardioinfantil de Bogotá, Colombia, entre el 26 marzo de 2020, fecha de inicio del protocolo de atención para pacientes con sospecha de infección por SARS-Cov-2 (código plateado), y el 8 de junio, cuando se inició el estudio. La recolección de los datos se realizó de forma retrospectiva en el periodo del 26 de marzo al 8 de junio, y desde esa fecha la recolección de los datos se hizo de forma prospectiva hasta el 30 de junio, fecha en la que acabó la recolección de los datos. Los pacientes incluidos fueron diagnosticados según el consenso colombiano de atención, diagnóstico y manejo de la infección por SARS-Cov-2 ^16^.

Se hizo un muestreo no probabilístico consecutivo de todos los pacientes con indicación de hospitalización que cumplían con los criterios de inclusión del estudio. Se consideró como caso confirmado a cualquier "persona con un cuadro clínico sospechoso o asintomática con un resultado positivo en alguna de las pruebas moleculares o genómicas que detectan SARS-CoV2/ COVID-19" (16, p. 8). Se excluyeron los pacientes que no tenían resultados de exámenes de laboratorio establecidos como factores pronósticos (gases arteriales, dímero D, ferritina, LDH, hemograma, troponina y creatinina).

Se recolectaron de forma retrospectiva los datos de antecedentes patológicos, síntomas, resultados de pruebas de laboratorio e imágenes diagnósticas, tratamiento, complicaciones y resultado final registrados entre el 26 de marzo y el 8 de junio de 2020 en la historia clínica electrónica de los pacientes; a partir de esa fecha, se recolectaron los datos concurrentemente a medida que se incluían los individuos en el estudio y hasta el 30 de junio de 2020. El seguimiento de los pacientes se extendió hasta el día del egreso hospitalario.

Se definieron dos grupos: el de casos graves, es decir, aquellos pacientes que requirieron soporte respiratorio mediante intubación orotraqueal, y el de casos no graves, constituido por los pacientes que no lo requirieron ^17^. Teniendo en cuenta que la probabilidad de sobrevivir en el grupo de casos no graves era del 98 % y en el grupo de casos graves del 80 % ^18^, se calculó un tamaño de muestra de 94 pacientes. Las características radiológicas y la definición de caso de COVID-19 se describen en el apéndice o material suplementario enviado a la revista, en los que se amplían algunas definiciones.

### Análisis estadístico

El análisis estadístico se hizo con el programa Stata 14.2™ de la siguiente manera.

### Estadística descriptiva

Se usó estadística descriptiva básica para las variables continuas (media con desviación estándar, moda con rango intercuartílico) y las variables categóricas (frecuencias y porcentajes) para caracterizar los pacientes según la gravedad de la infección y la mortalidad.

### Análisis bivariado

En las variables continuas, las comparaciones entre los casos graves y la mortalidad se hicieron mediante la prueba t de Student para las variables distribuidas normalmente y la prueba de Mann-Whitney para aquellas sin distribución normal, en tanto que para los datos nominales se utilizó la prueba de ji al cuadrado. La distribución de los datos se analizó mediante la prueba de Shapiro-Wilk, métodos gráficos, valores de simetría y medidas de curtosis.

### Análisis de supervivencia

La supervivencia global se analizó con el estimador de Kaplan-Meier estableciendo como evento de interés la mortalidad y como resultado secundario, la gravedad. Todos los pacientes tuvieron seguimiento complete y no hubo censuras. El tiempo hasta el evento correspondió a los dias transcurridos desde el inicio de la hospitalización hasta la muerte o el egreso. Las variables con valores de p<0,20 en el análisis bivariado se consideraron para el análisis multivariado.

### Análisis multivariado

La asociación entre el grupo de variables independientes con un valor de p<0,2 en la prueba de Mantel-Cox *(log rank)* y el tiempo hasta la muerte se evaluó mediante un modelo de regresión de Cox que después se redujo a uno más parsimonioso, proceso que se repitió para evaluar la gravedad. La evaluación del supuesto de riesgos proporcionales se hizo para cada modelo obtenido mediante los residuales de Schoenfeld.

## Resultados

**Figura 1:**
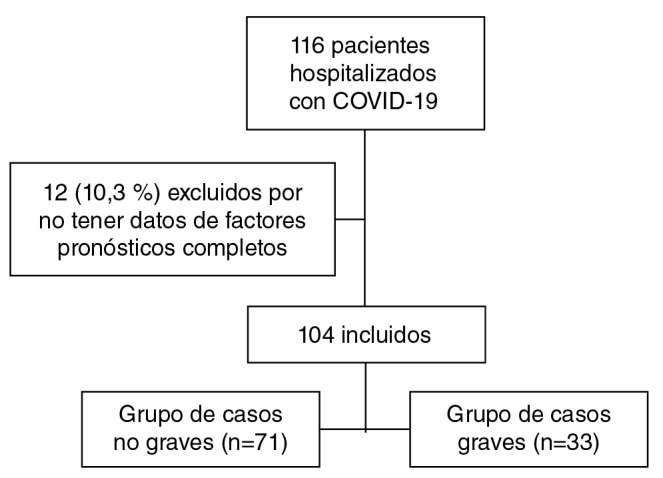
Diagrama de flujo de la selección de los pacientes incluidos en el estudio

Se incluyeron 104 pacientes que cumplían con los criterios de selección. En el 68,2 % (n=71), de ellos la infección no fue grave y en el 31,7 % (n=33), lo fue (figura 1). El 9,6 % (n=10) murió y de dicho porcentaje el 90 % (9 casos) tenía la infección grave.

### Características sociodemográficas y clínicas

En cuanto a la edad, la media fue de 59 años (desviación estándar, DE de 16,9) y se encontró una diferencia significativa entre los pacientes que murieron y los que sobrevivieron, con medias de 58,5 (DE de 16,8 y 73,1, respectivamente) (p=0,009). En cuanto al sexo, el 52,8 % correspondió a hombres (n=55) y no se encontraron diferencias significativas entre hombres y mujeres en la gravedad o la mortalidad. La media del índice de masa corporal (IMC) fue de 26,5 (DE=5,1) y no se observaron diferencias significativas para esta variable entre los grupos. En cuanto al grupo sanguíneo, la mayoría de los pacientes eran 0+ (62,5 %, n=65), y no se encontraron diferencias para la gravedad o la mortalidad. El 29,5 % de los pacientes tenía historia de tabaquismo o eran fumadores activos, aunque no hubo diferencias entre los grupos. La prevalência de hipertensión arterial fue de 53,8 % (n=56) y 8 de los 10 pacientes que murieron tenían antecedente de esta condición. El 28,8 % de los pacientes era diabético y 8 de los 10 que murieron tenían la enfermedad (p=0,000). La descripción detallada de las características sociodemográficas y clínicas de los individuos incluidos en el estudio se presenta en el cuadro 1.

**Cuadro 1:** Características sociodemográficas y clínicas de los pacientes incluidos en el estudio

Característica	Todos	Casos graves (n=71)	Casos no graves (n=33)	p	Sobrevivientes (n=94)	Fallecidos (n=10)	p
Edad (años), media (DE)	59,9 (16,9)	59,3 (18,0)	61,1 (14,6)	0,603	58,5 (16,8)	73,1 (11,8)	0,009*
Edad (años), n (%)							
<40	16 (15,3)	12 (16,9)	4 (12,1)	0,864	16 (17,0)	0	0,029
40-65	46 (44,2)	32 (45,07)	14 (42,4)	0,864	44 (46,8)	2 (20)	0,029
66-80	27 (25,9)	17 (23,9)	10 (30,3)	0,864	23 (24,4)	4 (40)	0,029
>80	15 (14,4)	10 (14,0)	5 (15,1)	0,864	11 (11,7)	4 (40)	0,029
Edad (años), n (%)				0,993			0,053
<60	51 (49,0)	35 (49,3)	16 (48,4)		49 (52,1)	2 (20)	
>60	53 (50,9)	36 (50,7)	17 (51,2)		45 (47,8)	8 (80)	
Sexo, n (%)				0,817			0,848
Hombre	55 (52,8)	37 (52,1)	18 (54,5)		50 (53,1)	5 (50)	
Mujer	49 (47,1)	34 (47,8)	15 (45,4)		44 (46,8)	5 (50)	
IMC, media (DE)	26,5 (5,1)	26,4 (4,8)	26,7 (5,8)	0,764	26,7 (5,0)	24,0 (5,0)	0,103*
IMC (kg/m2), n (%)							
<18,5	3 (2,8)	1 (1,4)	2 (6,0)	0,187	2 (2,1)	1 (10)	0,157*
18,5-24,9	41 (39,4)	31 (43,6)	10 (30,3)	0,194	36 (38,3)	5 (50)	0,472
25-29,9	34 (32,6)	24 (33,8)	10 (30,3)	0,723	31 (32,9)	3 (30,0)	0,849
>30	26 (25)	15 (21,1)	11 (33,3)	0,181	25 (26,6)	1 (10)	0,249
Grupo sanguíneo, n (%)				0,503			0,248
A+	29 (27,8)	22 (30,9)	7 (21,2)		28 (29,7)	1 (10)	
A-	1 (0,96)	1 (1,4)	0		1 (1,0)	0	
B+	7 (6,7)	6 (8,4)	1 (3,0)		6 (6,3)	1 (10)	
O+	65 (62,5)	41 (57,7)	24 (72,2)		58 (61,7)	7 (70)	
O-	2 (1,9)	1 (1,4)	1 (3,0)		1 (1,06)	1 (10)	
Tabaquismo, n (%)				0,608			0,644
Nunca fumó	70 (67,3)	50 (70,4)	20 (60,6)		63 (67,0)	7 (70)	
Historia de tabaquismo	29 (27,8)	18 (25,3)	11 (33,3)		27 (28,7)	2 (20)	
Fumador activo	5 (4,8)	3 (4,2)	2 (6,0)		4 (4,2)	1 (10)	
Antecedentes, n (%)							
Hipertensión arterial	56 (53,8)	38 (53,5)	18 (54,5)	0,922	48 (51,0)	8 (80)	0,081*
Diabetes	30 (28,8)	18 (25,3)	12 (36,3)	0,249	22 (23,4)	8 (80)	0,000*
Enfermedad coronaria	15 (14,4)	11 (15,4)	4 (12,1)	0,649	12 (12,7)	3 (30)	0,140*
Enfermedad renal crónica	11 (10,5)	7 (9,8)	4 (12,1)	0,727	9 (9,5)	2 (20)	0,308
EPOC	12 (11,5)	10 (14,0)	2 (6,06)	0,233	9 (9,5)	3 (30)	0,055*
Asma	5 (4,8)	0	5 (5,1)	0,001	4 (4,2)	1 (10)	0,419
EPID	1 (0,9)	1 (1,4)	0	0,493	1 (1,0)	0	0,743
Neoplasia sólida	9 (8,6)	6 (8,4)	3 (9,0)	0,914	7 (7,4)	2 (20)	0,179
Neoplasia hematológica	4 (3,8)	4 (5,6)	0	0,164	4 (4,2)	0	0,506
ACV	6 (5,7)	3 (4,2)	3 (9,0)	0,322	3 (3,1)	3 (30)	0,001*
Artritis reumatoide	4 (3,8)	2 (2,8)	2 (6,0)	0,423	3 (3,1)	1 (10)	0,287
Trasplante de riñón	1 (0,9)	1 (1,4)	0	0,493	1 (1,0)	0	0,743
Vasculitis	1 (0,9)	0	1 (3,0)	0,141	1 (1,0)	0	0,743
Cirrosis	1 (0,9)	1 (1,4)	0	0,493	0	1 (10)	0,002*
Trasplante de corazón	1 (0,9)	1 (1,4)	0	0,493	1 (1,0)	0	0,743
Lupus	2 (1,9)	1 (1,4)	1 (3,0)	0,575	1 (1,06)	0	0,743
Trasplante de hígado	1 (0,9)	1 (1,4)	0	0,493	1 (1,0)	0	0,743
Toma IECA o ARA	46 (44,2)	30 (42,2)	16 (48,8)	0,552	40 (42,5)	6 (60)	0,291
Falla cardiaca				0,761			0,975
40-50	1 (0,9)	1 (1,4)	0		1 (1,06)	0	
<40	8 (7,6)	6 (8,4)	2 (6,0)		7 (7,4)	1 (10)	
>50	9 (8,6)	7 (9,8)	2 (6,0)		8 (8,5)	1 (10)	
Hemodiálisis, n (%)	4 (3,8)	2 (2,8)	2 (6,0)	0,423	2 (2,1)	2 (20)	0,005*
Uso crónico de corticoides, n (%)	9 (8,6)	5 (7,0)	4 (12,1)	0,391	8 (8,5)	1 (10)	0,873
Uso de micofenolato, n (%)	5 (4,8)	5 (7,0)	0	0,118	5 (5,3)	0	0,455
Uso de tacrolimus, n (%)	6 (5,7)	6 (8,4)	0	0,085	6 (5,7)	0	0,410

DE: desviación estándar; IMC: índice de masa corporal; EPOC: enfermedad pulmonar obstructiva crónica; EPID: enfermedad pulmonar intersticial difusa; ACV: accidente cerebrovascular; IECA: inhibidores de la enzima convertidora de angiotensina; ARA: antagonistas de los receptores de la angiotensina

El 72,1 % (n=75) no había tenido contacto reconocido con personas contagiadas, el 23,1 % (n=24) había tenido contacto estrecho con una persona positivo para SARS-CoV-2 y el 4,8 % (n=5) correspondía a trabajadores de la salud. El síntoma más frecuente fue la tos (74,1 %, n=75), seguido de fiebre (66,7 %, n=70), disnea (62,9 %, n=66) y odinofagia (17,1%, n=18). Además, se detectaron síntomas gastrointestinales: diarrea (20 %, n=21), vómito (12,4 %, n=13) y náuseas (4 %, n=4), así como síntomas neurológicos: cefalea (17,1%; n=18), alteración de la conciencia (7,6 %, n=8), disgeusia (5,7 %, n=6), anosmia (4,8 %, n=5) y convulsiones (1 %, n=1).

### Características de la hospitalización

La hospitalización en la unidad de cuidados intensivos fue necesaria en 56 pacientes (53,8 %), con una media de estancia de cinco días (DE=7,2). Se encontró una diferencia significativa en el tiempo de estancia entre los pacientes con enfermedad grave y aquellos que no la presentaron, con medias de 12,9 (DE=7,7) y 1,4 días (DE=2,7), respectivamente (p=0,000). La media de tiempo con necesidad de respiración mecánica asistida fue de 3,7 días (DE=9,7) y fue más alta entre los fallecidos (8,8 días, DE=8,4; p=0,010). El 31,7 % de los pacientes presentó síndrome de dificultad respiratoria aguda, siendo esta variable un factor de riesgo significativo para la gravedad (p=0,000) y la mortalidad (p=0,001). Todos los pacientes que requirieron pronación y relajación (n=24; 23 %) pertenecían al grupo de casos graves (p=0,000) y, de ellos 5 murieron (p=0,034). Asimismo, todos los pacientes que requirieron uso de vasopresores (n=16, 15,3 %) pertenecían al grupo de los casos graves y, de estos, 7 fallecieron (p=0,000).

El 16,3 % de los pacientes (n=17) presentó choque séptico y el 24 % (n=25), lesión renal aguda, y de estos últimos, el 50 % murió. Todos los pacientes que requirieron terapia de reemplazo renal (4,8 %, n=5) fallecieron (p=0,000). Dos pacientes presentaron coagulación intravascular diseminada (1,9 %), pero ninguno falleció. Otras complicaciones observadas fueron la miocarditis (n=1), el choque cardiogénico (n=2), la tromboembolia pulmonar (n=6) y las infecciones asociadas con la atención en salud (n=16). La descripción de las variables hospitalarias se presenta en el cuadro 2.

**Cuadro 2:** Características hospitalarias de los pacientes incluidos en el estudio

Variable	Todos	Casos no graves (n=71)	Casos graves (n=33)	p	Sobrevivientes (n=94)	Fallecidos(n=10)	p
Días de estancia en UCI, media (DE)	5,0 (7,2)	1,4 (2,7)	12,9 (7,7)	0,000	4,7 (7,1)	8,6 (7,8)	0,107*
Días de respiración mecánica asistida, media (DE)	3,7 (9,7)	0,05 (8,6)	11,8 (9,5)	0,000	3,2 (9,8)	8,8 (8,4)	0,010*
SDRA, n (%)	33 (31,7)	2 (2,8)	31 (93,9)	0,000	25 (26,6)	8 (80)	0,001*
Pronación, n (%)	24 (23,0)	0	24 (72,7)	0,000	19 (20,2)	5 (50)	0,034*
Relajación, n (%)	24 (23,0)	0	24 (72,7)	0,000	19 (20,2)	5 (50)	0,034*
Vasopresor, n (%)	16 (15,3)	0	16 (48,4)	0,000	9 (9,5)	7 (70)	0,000*
Choque séptico, n (%)	17 (16,3)	16 (48,4)	1 (1,4)	0,000	7 (7,4)	10 (100)	0,000*
LRA, n (%)	25 (24,0)	12 (16,9)	13 (39,3)	0,001	20 (21,2)	5 (50)	0,000*
TRR, n (%)	5 (4,8)	1 (1,4)	4 (12,1)	0,001	7 (7,4)	10 (100)	0,000*
CID, n (%)	2 (1,9)	2 (6,0)	0	0,036	0	2 (20)	0,000*
Miocarditis, n (%)	1 (0,9)	0	1 (3,0)	0,141	1 (1,0)	0	0,743
Choque cardiogénico, n (%)	2 (1,9)	0	2 (6,0)	0,038	2 (2,1)	0	0,640
TEP, n (%)	6 (5,7)	1 (1,4)	5 (15,1)	0,005	5 (5,3)	1 (10)	0,546
Infección asociada con la atención en salud, n (%)				0,000			0,114*
Asociada a catéter	3 (2,8)	0	3 (9,0)		2 (2,1)	1 (10)	
Urinaria	5 (4,8)	3 (4,2)	2 (6,0)		4 (4,2)	1 (10)	
Neumonía asociada con respirador	6 (5,7)	0	6 (18,1)		4 (4,2)	2 (20)	
Neumonía adquirida en el hospital	2 (1,9)	1 (1,4)	1 (3,0)		2 (2,1)	0	
Días desde inicio de los síntomas, media (DE)	6,4 (4,1)	6,7 (4,4)	5,7 (3,4)	0,235	6,4 (4,0)	6,7 (5,5)	0,840
Puntaje NEWS en el ingreso, n (%)	5,6 (0,33)	4,2 (0,2)	8,6 (0,6)	0,000	5,1 (0,3)	10,6 (1,1)	0,000*
Riesgo bajo	0,4 (0,04)	0,5 (0,05)	0,09 (0,05)	0,000	0,46 (0,05)	0	0,004*
Riesgo medio	0,27 (0,07)	0,29 (0,05)	0,24 (0,07)	0,576	0,29 (0,04)	0,1 (0,1)	0,181
Riesgo alto	0,29 (0,04)	0,12 (0,03)	0,66 (0,08)	0,000	0,23 (0,04)	0,9 (0,1)	0,000*
Puntaje SOFA en el ingreso, media (DE)	3,3 (0,2)	2,8 (0,3)	4,5 (0,48)	0,002	3,0 (0,2)	6,2 (0,9)	0,000*
Signos vitales en el ingreso, media (DE)							
Frecuencia respiratoria	21,0 (8,9)	19,2 (3,2)	25 (14,6)	0,002	20,8 (9,0)	23 (9,0)	0,477
SO_2_	86,1 (9,9)	87,9 (6,7)	82,5 (14,0)	0,009	86,1 (10,2)	87 (5,2)	0,787
Frecuencia cardiaca	95 (19,2)	93,3 (19,8)	98,7 (17,4)	0,182	94,9 (18,8)	96,3 (23,2)	0,835
Tensión sistólica	123,8 (24,2)	124,2 (24,1)	123,0 (24,8)	0,814	124,4 (23,7)	117,9 (29,6)	0,416
Tensión diastólica	72,0 (14,8)	73,0 (14,1)	69,8 (16,2)	0,311	72,5 (13,7)	67,8 (23,3)	0,343
Tensión media	89,2 (16,3)	89,9 (15,7)	87,7 (17,7)	0,531	89,6 (15,4)	85,3 (24,3)	0,426
Temperatura	36,9 (0,9)	36,8 (0,1)	37,1 (0,19)	0,085	36,9 (0,1)	36,9 (0,3)	0,898
Signos vitales durante hospitalización, media (DE)							
Frecuencia respiratoria	19,6 (6,3)	20,0 (7,2)	18,6 (4,0)	0,279	19,7 (6,6)	18,1 (2,3)	0,436
SO_2_	92,0 (8,1)	92,1 (9,6)	91,6 (3,5)	0,773	92,1 (8,5)	91,1 (3,3)	0,704
Frecuencia cardiaca	82,3 (16,9)	81,2 (17,1)	84,7 (16,4)	0,327	81,9 (16,3)	86,4 (21,9)	0,430
Tensión sistólica	113,2 (17,1)	112 (17,6)	113,9 (16,3)	0,787	112,8 (16,7)	117,7 ( 21,4)	0,396
Tensión diastólica	65,9 (11,4)	66,3 (11,9)	65,0 (10,4)	0,602	66,3 (11,6)	62,2 ( 9,0)	0,281
Tensión media	81,2 (11,5)	81,2 (12,0)	81,3 (10,6)	0,983	81,3 (11,6)	80,8 (11,8)	0,893
Temperatura	36,7 (0,7)	36,7 (0,9)	36,7 (0,1)	0,996	36,7 (0,7)	36,7 (1,3)	0,928

CID: coagulación intravascular diseminada; TEP: tromboembolia pulmonar; NEWS: *National Early Warning Score;* SOFA: *Sequential Organ Failure Assessment*

### Características radiológicas y paraclínicas

El patrón radiológico típico de la COVID-19 se evidenció en 62 pacientes (69,6 %): 79 con patrón radiológico en vidrio esmerilado (75,9 %); 30 con patrón en empedrado (28,8 %), y en 3 se observó el signo del halo invertido (2,8 %). No se encontró una relación entre estos hallazgos y la gravedad o la mortalidad. De los 34 (32,9 %) pacientes que tuvieron consolidación, 20 (19,4 %) presentaron el patrón clásico de la COVID-19; 3 (2,9 %), uno diferente, y 11 (10,6 %) se consideraron hallazgos indeterminados. Se encontró una asociación significativa entre la consolidación y la mortalidad (p=0,004). Se observaron nódulos aleatorios en 5 pacientes (4,8 %) y anormalidades intersticiales en 10 (9,5 %). Los resultados radiológicos y paraclínicos se presentan en el cuadro 3.

**Cuadro 3:** Características radiológicas y de laboratorio de los pacientes incluidos en el estudio

Variable	Todos	Casos no graves (n=71)	Casos graves (n=33)	p	Sobrevivientes (n=94)	Fallecidos (n=10)	p
Patrón radiológico, n (%)				0,850			0,189*
No tiene TC	23 (22,1)	17 (23,9)	6 (18,1)		21 (22,3)	2 (20)	
Patrón típico	62 (59,6)	42 (59,1)	20 (60,6)		58 (61,7)	4 (40)	
Indeterminado	16 (15,3)	10 (14,0)	6 (18,1)		13 (13,8)	3 (30)	
No COVID-19	2 (1,9)	1 (1,4)	1 (3,0)		1 (1,0)	1 (10)	
Normal	1 (0,9)	1 (1,4)	0		1 (1,0)	0	
Vidrio esmerilado, n (%)	79 (75,9)	53 (74,6)	26 (78,7)	0,643	72 (76,6)	7 (70)	0,643
Patrón en empedrado, n (%)	30 (28,8)	19 (26,7)	11 (33,3)	0,491	28 (29,7)	2 (20)	0,516
Signo halo inverso, n (%)	3 (2,8)	1 (1,4)	2 (6,0)	0,187	3 (3,1)	0	0,566
Consolidación, n (%)				0,987			0,004*
Patrón no COVID-19	3 (2,9)	2 (2,8)	1 (3,0)		1 (1,0)	2 (20)	
Patrón clásico	20 (19,4)	14 (20)	6 (18,1)		18 (19,3)	2 (20)	
Patrón indeterminado	11 (10,6)	7 (10)	4 (12,1)		9 (9,6)	2 (20)	
Nódulos aleatorios, n (%)							
Patrón no COVID-19	5 (4,8)	3 (4,2)	2 (6,0)	0,684	4 (4,2)	1 (10)	0,419
Intersticial, n (%)				0,720			0,001*
Patrón no COVID-19	6 (5,7)	4 (5,6)	2 (6,0)		3 (3,1)	3 (30)	
Patrón indeterminado	4 (3,8)	2 (2,8)	2 (6,0)		3 (3,1)	1 (10)	
Exámenes de laboratorio, media (DE)							
PO_2_	65,0 (17,6)	64,3 (13,1)	66,5 (24,8)	0,559	64,1 (16,2)	73,7 (27,0)	0,103*
pCO_2_	35,2 (11,4)	34,6 (11,4)	36,6 (11,3)	0,413	35,2 (11,9)	34,9 (4,9)	0,938
PAFI	236,3 (68,1)	258,4 (53,6)	188,7 (72,4)	0,000	241,5 (63,8)	186,9 (89,9)	0,015*
Lactato	3,9 (21,9)	4,7 (26,5)	2,0 (1,1)	0,555	4,1(23,0)	2,0 (0,9)	0,780
Leucocitos	8275 (4297)	7665 (3656)	9588 (5252)	0,032	7920 (3829)	11614 (6790)	0,009*
Neutrófilos	6243 (4034)	5674 (3661)	7467 (4560)	0,034	5920 (3725)	9283 (5613)	0,011*
Linfocitos	1209 (592)	1275 (631)	1065 (474)	0,091	1195 (582)	1333 (700)	0,485
Plaquetas	231706 (89976)	233739 (87670)	227333 (96000)	0,737	235675 (90483)	194400 (79621)	0,169*
Hemoglobina	14,5 (2,8)	14,3 (2,6)	14,8 (3,2)	0,399	14,7 (2,6)	12,5 (4,1)	0,023*
AST	56,6 (56,3)	50,0 (41,9)	69,8 (76,8)	0,104	50,4 (40,6)	109,4 (120,5)	0,001*
ALT	54,2 (54,0)	56,6 (60,8)	49,5 (37,2)	0,546	55,3 (55,7)	44,9 (36,1)	0,565
Bilirrubina total	0,88 (0,8)	0,91 (0,9)	0,8 (0,5)	0,693	0,8 (0,8)	0,9 (0,5)	0,959
Bilirrubina directa	0,46 (0,5)	0,46 (0,6)	0,44 (0,3)	0,865	0,46 (0,5)	0,43 (0,2)	0,851
LDH	386,8 (170)	344,1 (147,5)	476,1 (184,0)	0,000	374,7 (166,4)	512,4 (175,5)	0,020*
Troponina	3,7 (36,5)	5,4 (44,0)	0,04 (0,1)	0,505	4,1 (38,3)	0,12 (0,2)	0,755
Dímero D	16,1 (80,5)	21,7 (96,2)	3,3 (5,2)	0,299	17,2 (83,9)	3,3 (3,7)	0,641
Creatinina	1,1 (1,0)	1,1 (0,8)	1,2 (1,0)	0,697	1,0 (0,77)	1,8 (1,7)	0,018*
BUN	19,5 (1,3)	20 (15,4)	18,5 (8,3)	0,606	19,1 (136)	23,4 (10,1)	0,346
Ferritina	1450 (1711)	1101 (1245)	2235 (2298)	0,003	1341 (1334)	2760 (4140)	0,034*
Sodio	103,1 (10,8)	101,9 (5,4)	105,6 (10,8)	0,099	103,2 (11,2)	102 (5,5)	0,728
Potasio	4,1 (0,6)	4,0 (0,5)	4,3 (0,6)	0,048	4,1 (0,5)	4,1 (0,5)	0,972
Glucosa	115,4 (46,2)	110,8 (42,6)	125,1 (52,7)	0,143	113,4 (45,7)	134,2 (49,2)	0,178
PCR	10,1 (8,8)	6,8 (6,6)	16,5 (9,1)	0,000	9,1 (8,1)	18,9 (10,3)	0,000*

TAC: tomografía computarizada; COVID-19: *Coronavirus disease* 2019; DE: desviación estándar; PAFI: PaO_2_/FiO_2_; AST: aspartato aminotransferasa; ALT: alanina aminotransferasa; LDH: deshidrogenasa láctica; BUN: nitrógeno ureico en sangre; PCR: proteína C reactiva

### Uso de medicamentos

De los 26 pacientes que recibieron hidroxicloroquina, 12 eran casos graves y 14 no lo eran; 2 de los pacientes que recibieron este tratamiento fallecieron. De los 10 pacientes que fallecieron, 2 presentaron prolongación del intervalo QT; del grupo total, 8 (7,6%) presentaron esta alteración del ritmo cardiaco y 4 de ellos habían recibido tratamiento con hidroxicloroquina. El 55,7 % de los pacientes recibió tratamiento con claritromicina (n=58) y se encontró una relación significativa entre el uso de este medicamento y la gravedad de la enfermedad, ya que el 75,7 % de los pacientes con infección grave lo recibieron (p=0,005); 23 (24,2 %) pacientes recibieron tratamiento con corticoide y no se observó una relación entre su uso y la gravedad o la mortalidad. La información sobre el uso de medicamentos se presenta en el cuadro 4.

**Cuadro 4:** Uso de medicamentos en los pacientes incluidos en el estudio

Variable	Todos	Casos no graves (n=71)	Casos graves (n=33)	P	Sobrevivientes (n=94)	Fallecidos (n=10)	P
Medicamentos, n (%)							
Hidroxicloroquina	26 (25)	14 (19,7)	12 (36,3)	0,068	24 (25,5)	2 (20)	0,701
Claritromicina	58 (55,7)	33 (46,4)	25 (75,7)	0,005	51 (54,2)	7(70)	0,341
Corticoides				0,359			0,261
Dexametasona	18 (19,3)	13 (18,5)	5 (15,6)		18 (19,3)	0	
Prednisolona	5 (4,9)	2 (2,8)	3 (9,3)		4 (4,3)	1 (11,1)	

### Análisis de la mortalidad

**Figura 2:**
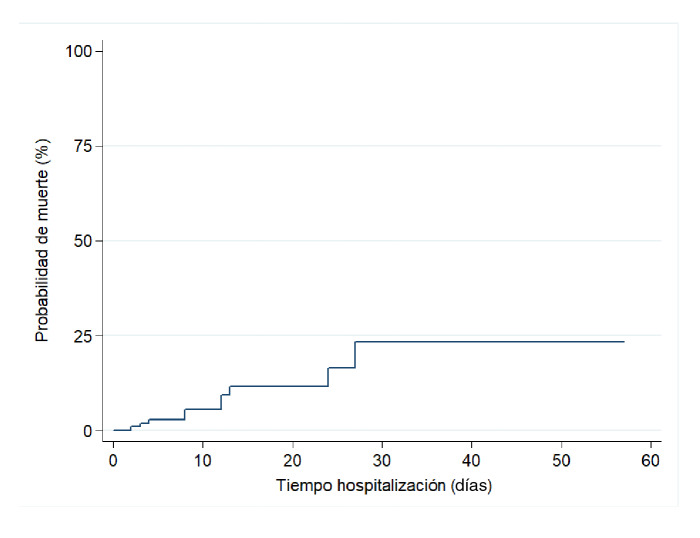
Probabilidad de muerte en toda la cohorte

**Figura 3:**
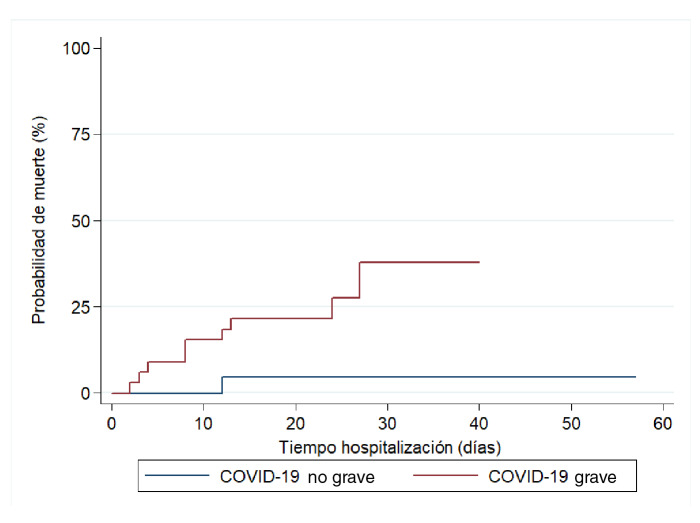
Probabilidad de muerte según la gravedad de la enfermedad

La incidencia acumulada de muerte fue de 9,6 %. El tiempo de hospitalización fluctuó entre 2 y 57 días, y los 104 pacientes acumularon un total de tiempo en riesgo de 1.403 días. Con 10 fallecimientos en la cohorte, se estimó una tasa de mortalidad de 0,69 por 100 pacientes-día (figura 2). Al comparar los grupos se encontró que la probabilidad de muerte durante la hospitalización en el grupo de casos graves fue de 37,9 %, en tanto que en el de casos no graves fue de 4,7 % (figura 3).

Para el análisis multivariado se tomaron las variables con p<0,2 en el análisis previo y se tuvo en cuenta la plausibilidad biológica para incluirlas en el modelo, de manera que en un primer modelo se incluyeron las siguientes variables: gravedad, edad, IMC, hipertensión arterial, diabetes, enfermedad coronaria, accidente cerebrovascular, lesión renal aguda, coagulación intravascular diseminada, infecciones asociadas con la atención en salud, consolidación y presión parcial de dióxido de carbono (pC0_2_). A partir de este modelo, y con el fin de tener uno más parsimonioso, se sacaron las variables con los valores más altos de p una a una y se dejaron solo aquellas con significación estadística. Se encontró que la gravedad aumentó el riesgo de mortalidad 21,2 veces (p=0,003), la edad mayor de 60 años, 13,5 veces (p=0,014), el IMC menor de 18, 21 veces (p=0,009), tener lesión renal aguda, 1,9 veces (p=0,014) y tener consolidación, 5,8 veces (p=0,010). No se encontró violación del supuesto de riesgos proporcionales en la evaluación de los residuales de Schoenfeld (p=0,91) (cuadro 5).

**Cuadro 5:** Factores asociados con la mortalidad por COVID-19

Variable	HR	IC_95%_	P	Evaluación del supuesto de riesgos proporcionales
Gravedad	21,2	2,2-197.5	0,003	0,8782
Edad>60 años	13,5	1,7-104,9	0,014	0,6300
IMC<18	21,0	1,2-344,0	0,009	0,9489
LRA	1,9	0,9-4,1	0,014	0,9415
Consolidación	5,8	1,4-24,0	0,010	0,4428
				Prueba global: 0,9102

HR: *hazard ratio;* IC: intervalo de confianza; IMC: índice de masa corporal; LRA: lesión renal aguda

### Análisis de la gravedad

**Figura 4:**
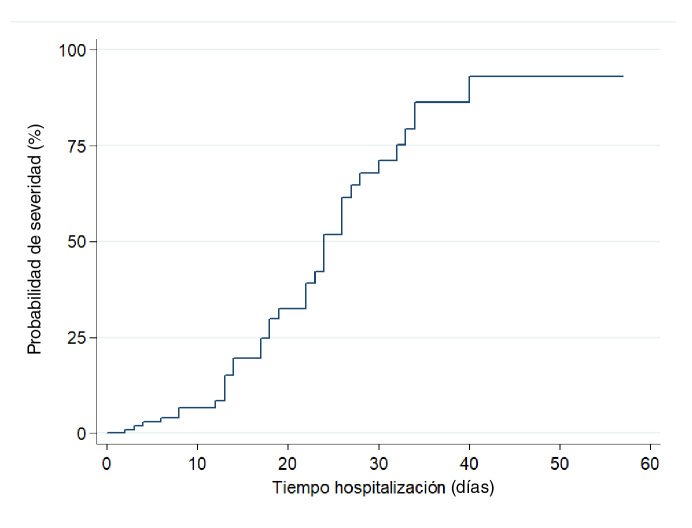
Probabilidad de gravedad

Se encontraron 33 casos graves y la incidencia acumulada de gravedad fue de 31,7 %. Teniendo en cuenta que el tiempo en riesgo de los 104 pacientes fue de 1.403 días, se estimó una tasa de gravedad en la cohorte de 2,3 por 100 pacientes-día (figura 4).

Para el análisis multivariado se tomaron las variables con p<0,2 en el análisis previo y se tuvo en cuenta la plausibilidad biológica para incorporarlas en el modelo, de manera que en un primer modelo se incluyeron las variables de edad, asma, diabetes, uso de tacrolimus, uso de micofenolato, hemodiálisis, infección asociada con el cuidado de la salud, puntaje NEWS2 en el momento del ingreso, puntaje del *Sequential Organ Failure Assessment* (SOFA) en el momento del ingreso, frecuencia cardiaca, dióxido de azufre (SO_2_), temperatura en el momento del ingreso, frecuencia respiratoria en el momento del ingreso, índice PAFI, leucocitos, neutrófilos, linfocitos, LDH, ferritina, sodio, potasio, glucosa y proteína C reactiva.

A partir de este modelo, y con el fin de tener uno más parsimonioso, se excluyeron las variables con los valores más altos de p una a una y se dejaron solo aquellas con significación estadística. Se encontró que las variables asociadas con el riesgo de gravedad fueron la hemodiálisis (HR=135,6; p=0,000), la diabetes (HR =4,4; p=0,002), la LDH (HR=1,004; p=0,000), el puntaje NEWS2 en el ingreso (HR=1,15; p=0,010) y los leucocitos (HR=1,002; p=0,000). Se determinó que los linfocitos eran un factor protector frente a la gravedad (HR=0,99; p=0,001). No se encontró violación del supuesto de riesgos proporcionales en la evaluación de los residuales de Schoenfeld (p=0,99) (cuadro 6).

**Cuadro 6:** Factores asociados con la gravedad de la COVID-19

Variable	HR	IC_95%_	P	Evaluación del supuesto de riesgos proporcionales
Hemodiálisis	135,6	11,4-16,01	0,000	0,6388
Diabetes	4,4	1,7-11,5	0,002	0,7734
Linfocitos	0,99	0,996-0,999	0,001	0,8348
LDH	1,004	1,002-1,006	0,000	0,9335
Puntaje en NEWS al ingreso	1,15	1,03-1,28	0,010	0,7027
PCR	1,04	0,99-1,09	0,055	0,8305
Leucocitos	1,002	1,001-1,003	0,000	0,6597
				Prueba global: 0,9902

HR: hazard ratio; IC: intervalo de confianza; LDH: deshidrogenasa láctica; NEWS: *National Early Warning Score;* PCR: proteína C reactiva

## Discusión

El presente estudio es el primero que evalúa en Colombia las características clínicas de pacientes con diagnóstico de infección por SARS-Cov-2 y los factores relacionados con el desarrollo de la enfermedad grave y la mortalidad. Se encontró que cerca de una tercera parte de los pacientes hospitalizados cursó con enfermedad grave, similar a lo reportado por Li, ef *al.* y Wu, ef *al.*^18^^,^^19^, sin embargo, la mortalidad encontrada fue cercana al 20 %, menor que lo reportado en otros estudios ^10^^,^^14^^,^^15^^,^^20^. Un factor pronóstico bien establecido como la edad fue similar a lo reportado previamente ^2^^,^^10^^,^^15^^,^^18^: las personas de mayor edad son frágiles, tienen mayor carga de comorbilidades y un pronóstico menos favorable; en el estudio, a pesar de tener un porcentaje similar de personas mayores de 65 años, como ya se dijo, la mortalidad fue menor, lo que podría explicarse porque hasta el momento del estudio no se había superado la capacidad de camas disponibles en unidades de cuidados intensivos en la ciudad, lo que permitiría sugerir que la disponibilidad de camas en las unidades de cuidados intensivos sería un factor relacionado con una menor mortalidad. A diferencia de otros estudios, no se determinó el tabaquismo como un factor de riesgo de progresión de la enfermedad ^10^^,^^14^^,^^15^.

En cuanto a la sintomatología, dos terceras partes de los pacientes tenía fiebre al ingresar a urgencias, hallazgo llamativo que difiere del de otros estudios en las que se ha reportado un porcentaje cercano al 90 % ^1^^,^^2^^,^^10^^,^^11^. En el resto de los síntomas los porcentajes fueron similares a lo reportado en otros estudios ^1^^,^^2^^,^^10^^,^^11^^,^^18^, por lo que se puede inferir que la presencia de fiebre no tiene la misma importancia para el diagnóstico ni el manejo hospitalario en nuestra población, pero que la ausencia de este síntoma no descarta la enfermedad. Por otro lado, el porcentaje de síntomas gastrointestinales fue importante: 37 % de los pacientes los presentaron, dato de importancia para sospechar la presencia de la COVID-19, incluso en ausencia de síntomas respiratorios.

No se encontraron diferencias en los resultados relacionadas con el grupo sanguíneo. Si bien en estudios previos se ha asociado el grupo A con un mayor riesgo de infección ^21^^,^^22^, ello no se consideró un factor pronóstico en la población del estudio.

Entre las comorbilidades con significación estadística frente a la mortalidad aparece la diabetes mellitus en primer lugar, la cirrosis hepática, el accidente cerebrovascular y la hipertensión arterial crónica; sin embargo, no se observó una asociación entre la mortalidad y el uso de inhibidores de la enzima convertidora de angiotensina o los antagonistas de los receptores de la angiotensina II (ARA II), por lo cual se considera que los pacientes no deben suspender estos medicamentos porque el riesgo de afectar el control de la enfermedad de base sería mayor.

Por otra parte, el antecedente de asma y la hemodiálisis se asociaron con el desarrollo de la enfermedad grave, lo que no ha tenido la misma importancia en otros estudios ^10^^,^^14^. Por ello los pacientes con estos antecedentes, incluso en ausencia de otros factores de mal pronóstico, deben vigilarse con cuidado debido al riesgo de tener peores resultados finales.

No se encontró asociación entre un peor resultado final de la enfermedad y los antecedentes de cáncer, enfermedad autoinmunitaria, enfermedad pulmonar obstructiva crónica o trasplante de órgano sólido ni con la toma de inmunosupresores, variables que no habían sido estudiadas en detalle en estudios previos ^1^^,^^2^^,^^10^^,^^11^ y cuya poca frecuencia en este podría ser insuficiente para evaluar su valor pronóstico.

En cuanto a la obesidad, que se ha descrito como un factor independiente de mal pronóstico ^23^, no se encontró asociación con un peor resultado final, aunque la mortalidad sí fue mayor en pacientes con un IMC<18,5 kg/m^2^, lo que sugiere que el mal pronóstico estaría ligado al peso extremo y no solo a la obesidad. El punto de corte del IMC para la obesidad en otros estudios fue de 35 kg/m^2 (^^23^, en tanto que en este se estableció a partir de los 30 kg/m^2^, y no se hizo un análisis por grados de obesidad, lo que explicaría que no se haya determinado la obesidad como factor pronóstico en esta cohorte.

El puntaje NEWS2, que valora parámetros clínicos en el momento del ingreso, tuvo una excelente correlación con el resultado clínico, con una significación estadística para discriminar y predecir los resultados finales en los grupos de bajo y alto riesgo, en tanto que el puntaje SOFA tuvo significación estadística, con un punto de corte mayor de 6 puntos para predecir la mortalidad. Ello permite concluir que el NEWS2 debe realizarse en todos los pacientes para determinar su riesgo de mortalidad y enfermedad grave ^24^.

En cuanto a los resultados de los exámenes de laboratorio, la linfopenia no se asoció con un mayor riesgo de enfermedad grave ni con la mortalidad, por el contrario, el conteo de linfocitos mayor de 1.100 cél/µl fue un factor protector frente a la enfermedad grave ^1^^,^^2^^,^^10^^,^^11^. Otros resultados asociados con la mortalidad y la gravedad fueron la elevación de las enzimas hepáticas por encima de 100, el aumento de la LDH (>470 UI/L) y de la proteína C reactiva, con lo cual se plantea el posible beneficio de cambiar los puntos de corte descritos en otros estudios ^13^^,^^15^^,^^16^^,^^20^, hipótesis que debe corroborarse en estudios multicéntricos en la población colombiana con un mayor tamaño de muestra.

El patrón radiológico en la tomografía de tórax más frecuente fue el clásico para la COVID-19: patrón de vidrio esmerilado en 76 % de los pacientes, sobre todo en pacientes con sospecha de infección, en quienes la probabilidad previa a la prueba de cursar con la enfermedad aumentó en un porcentaje similar a lo descrito en otros estudios ^25^^-^^27^.

Las complicaciones más importantes fueron el desarrollo de síndrome de dificultad respiratoria aguda, el choque séptico y la lesión renal aguda, con una asociación estadísticamente significativa para la mortalidad, lo que los convierte en factores pronósticos importantes dado que su desarrollo durante la atención de los pacientes debe alertar al médico sobre los peores resultados finales, lo cual concuerda con lo descrito en otros estudios ^14^^,^^28^^,^^29^.

Con respecto al tratamiento, no se observaron diferencias en la mortalidad ni en el desarrollo de la enfermedad grave con el uso de la hidroxicloroquina o la claritromicina, pero sí hubo pacientes que los recibieron y presentaron prolongación significativa del intervalo QT. En concordancia con otros estudios, la ausencia de beneficio con estos fármacos ^30^ y el hallazgo de manifestaciones de toxicidad sugieren que no deben considerarse como opciones terapéuticas para la COVID-19. Tampoco se encontró beneficio en el uso de esteroides, a diferencia de lo reportado por otros autores ^31^, lo que se debería al bajo porcentaje de los pacientes que los recibieron en el presente estudio (24 %), pues los datos sobre su utilidad, principalmente de la dexametasona, se publicaron cuando este estudio ya había avanzado.

Una fortaleza de esta cohorte es el análisis de la mayoría de los factores asociados con la mortalidad y la gravedad en pacientes hospitalizados por COVID-19, así como la inclusión de aquellos con comorbilidades no estudiadas previamente o con reportes aún controversiales acerca del verdadero riesgo de un resultado adverso, como es el caso de personas con trasplante de órgano sólido. Entre las limitaciones cabe mencionar que se trató de un estudio en un solo centro, lo cual podría limitar la extrapolación de resultados a una población específica, además de que el tamaño muestral podría ser insuficiente para demostrar diferencias en ciertas variables.

No se incluyeron pacientes atendidos ambulatoriamente, pues no respondía al objetivo del estudio, caracterizado como observacional y sin muestreo aleatorio, pues incluyó a todos los pacientes que consultaron y cumplían con los criterios de hospitalización. Esto no permite proponer hipótesis sobre los pacientes ambulatorios ni conocer las implicaciones de la selección en el análisis de los resultados.

Deben hacerse estudios multicéntricos con un tamaño de muestra mayor para confirmar los hallazgos descritos en este y, además, validar otros factores de riesgo como la obesidad, el tabaquismo y la inmunosupresión por diferentes causas en la población del país.

La infección por SARS-Cov-2 (COVID 19), ha generado un impacto significativo en la morbimortalidad global, por lo que es necesario conocer los factores asociados con la gravedad y la muerte para priorizar la atención, enfocar el seguimiento y racionalizar los recursos. En este estudio las comorbilidades asociadas con una mayor mortalidad fueron la diabetes, la hipertensión arterial, la enfermedad renal crónica en hemodiálisis, la desnutrición y la cirrosis. Los resultados de exámenes paraclínicos asociados con un mal pronóstico fueron una LDH mayor de 470 UI/L y leucocitosis.

Como dato novedoso, el conteo de linfocitos superior a 1.100 cél/µl fue un factor protector. La mejor escala pronóstica fue el NEWS2, la cual debe usarse sistemáticamente en todos los pacientes para evaluar el pronóstico y definir el lugar de atención.
